# Trait-Specific Patterns of Phenotypic Differentiation Among Populations After Two Generations of Common-Garden Cultivation in a Wind-Pollinated Grass

**DOI:** 10.3390/plants15172556

**Published:** 2026-08-22

**Authors:** Hilda Meso Odongo, Melinda Halassy, Anna Mária Csergő, Katalin Török

**Affiliations:** 1 Doctoral School of Horticulture, Hungarian University of Agriculture and Life Sciences, 1118 Budapest, Hungary; 2 HUN-REN Centre for Ecological Research, Restoration Ecology Research Group, Institute of Ecology and Botany, Alkotmány u. 2-4, 2163 Vácrátót, Hungary; halassy.melinda@ecolres.hu (M.H.); torok.katalin@ecolres.hu (K.T.); 3 Department of Botany, Hungarian University of Agriculture and Life Sciences, 1118 Budapest, Hungary; csergo.anna.maria@uni-mate.hu

**Keywords:** common garden experiment, dry grassland, second generation, seed germination, seedling traits, seed transfer zone

## Abstract

Seed transfer guidelines are used in ecological restoration to reduce the maladaptation risk from non-local genotypes. Researchers often examine second-generation populations under uniform conditions to isolate adaptation from environmental responses. However, in outcrossing species, interpreting these traits may be challenging if uncontrolled gene flow and recombination among provenances influence offspring phenotypes. We compared seed germination and seedling traits of the wind-pollinated grass *Festuca vaginata*, a dominant species of open sand grasslands in Hungary, across seed transfer zones (STZs) and localities. We used a two-generation common garden experiment with uncontrolled gene flow. Our results reveal that under common garden cultivation, the transgenerational stability of population differentiation is traitspecific. While locality effects on germination disappeared in the second generation, phenotypic variation in biomass and leaf length persisted, suggesting that shared environments homogenize germination faster than vegetative traits. As wind-pollinated species often exhibit weak regional genetic structuring, STZs may not capture the primary axis of phenotypic variation in specific traits. While not invalidating the use of STZs, our findings suggest that seed sourcing strategies should, if feasible, consider locality-level variation when using wind-pollinated grasses for species reintroduction.

## 1. Introduction

The demand for native seeds for grassland restoration is increasing globally and across Europe, driven by initiatives such as the UN Decade on Ecosystem Restoration [1], the Convention on Biological Diversity post-2020 targets [2], and the European Nature Restoration Regulation [3,4]. The limited availability of native seeds represents a major practical bottleneck for large-scale restoration projects worldwide [5,6]. One reason for the lack of sufficient propagules is the need to consider provenance and use local seeds as much as possible [7,8]. To translate local provenance into practical guidelines, native seed transfer is often governed by Seed Transfer Zones (hereafter STZs) as a general proxy for multiple species [9,10]. STZs are geographically distinct areas within which seeds may be moved with minimal risk of maladaptation [11,12]. Some countries have developed regionally based seed-sourcing frameworks grounded in environmental and vegetation data rather than administrative boundaries [12]. Such frameworks aim to make seed transfer boundaries more biologically meaningful by reflecting patterns of genetic differentiation across ecological gradients, thereby reducing the risk of maladaptation between seed sources and restoration sites [13,14,15].

Common garden experiments are widely used to examine the adaptive versus plastic nature of trait variation among regions and source populations by growing different provenances under uniform environmental conditions [16,17]. Adaptation to the local environment is assessed by measuring fitness-related phenotypic traits [18] like biomass, plant height and seed mass, which directly influence survival and reproduction [19]. While local adaptation is known to enhance plant survival at restored sites [20], increased fitness may also result from phenotypic variation arising through immediate plastic responses to the environment, or, in subsequent generations, environmentally induced changes in progeny phenotype without underlying genetic divergence [16,21,22]. Distinguishing between these mechanisms is crucial, because evidence of plastic environmental responses may justify greater flexibility in seed sourcing under limited seed availability. When genetic differentiation among STZs is low, as found in some outcrossed and widely dispersed plant species with high natural levels of gene flow [15,23], moving seed sources across regions is less likely to cause maladaptation [15]. As a result, restoration efforts may draw on seeds from multiple provenances.

Studying trait expression across generations in common gardens is critical for understanding longer-term, transgenerational effects of origin and for modelling the ecological dynamics of restoration translocations [24]. However, interpreting patterns across generations presents several challenges. First, experimental designs often focus on exposing populations to a common environment [25] rather than preventing gene flow among flowering populations. This issue may be particularly relevant in outcrossing, wind-pollinated species, where extensive pollen dispersal can make reproductive isolation difficult to maintain. While reduced trait variation in offspring may indicate maternal environmental effects [26,27], unimpeded gene flow could also contribute to it via genetic swamping, inbreeding depression, outbreeding depression or heterosis [28,29]. On the other hand, persistent differences among provenances may indicate genetically based differentiation among populations [30]. In comparisons between F_0_ and F_1_ generations, however, observed trait differences may also reflect processes associated with seed multiplication, including genetic drift or sampling effects arising when only a subset of F_0_ individuals contributes to the subsequent generation [31]. Further, different traits may vary in their propensity to exhibit plastic versus genetically based responses to environmental variation [32]. Consequently, differentiation among populations may disappear rapidly for some traits while persisting across generations for others, highlighting the importance of simultaneously examining multiple fitness-related and functional traits. While these difficulties complicate the interpretation of trait variation across generations, they also mirror the demographic and genetic changes expected when novel populations are established from multiple source populations. Thus, second-generation common gardens provide a valuable framework for examining the levels and patterns of variation that may arise in restored populations, which to date have remained relatively understudied [33,34].

We selected *Festuca vaginata*, a wind-pollinated Pannonian sand grassland species of Hungary, to investigate whether and how the local trait variation in reproductive and vegetative traits observed in first-generation plants changes in their progeny. We focused on early plant life stages, which are particularly sensitive to the environmental changes experienced by organisms upon reintroduction for restoration [35]. Early life stages, like seed germination and seedling emergence, determine initial establishment success and hence are crucial for local adaptation [36,37]. We harvested seeds from the first-generation individuals grown in a common garden experiment in a study on local adaptation carried out by Cevallos et al. [38]. Because that study did not find any significant regional-level variation across STZs in the first generation, with most variation being concentrated at the local level, the authors concluded that relaxed seed sourcing can be used for restoration between STZs. Departing from the assumption that in wind-pollinated species the challenge associated with gene flow in common gardens may be minor due to the high natural levels of gene flow between populations and the weak geographic structuring of trait variation [39], genetic mixing was left uncontrolled in the first common garden. Therefore, growing seeds produced by first-generation plants in a second common garden enabled us to mimic a restoration experiment with mixed provenances. We explored the consequences of the new levels of variation for the novel population.

We tested the following hypotheses: (i) any phenotypic differentiation previously observed among STZs and localities would weaken after one additional generation under common garden conditions, and (ii) one generation under common garden conditions would modify patterns of phenotypic differentiation among germination and seedling traits.

## 2. Methods

### 2.1. Study Species

*Festuca vaginata* Waldst. and Kit. (Poaceae) is a wind-pollinated, drought-tolerant, perennial caespitose grass endemic to the Pannonian biogeographic region of Eastern Europe and adapted to arid environments. It exhibits short-distance, phalanx-type clonal growth [40], making it a dominant species in Pannonian sandy grasslands [41,42]. *F. vaginata* grasslands are of high conservation value due to their unique biodiversity and ecological characteristics.

### 2.2. Study Area

The seeds for the study were previously collected from open sand grasslands in Hungary, which belong to Pannonic inland dunes (Natura 2000 code: 2340) and Pannonic sand steppes (Natura 2000 code: 6260). These grasslands are among the most species-rich habitats of the Pannonian biogeographic region in Europe, harbouring exceptional biodiversity [43,44]. They have been extensively reduced and degraded by habitat fragmentation, invasion by alien species, urbanization, afforestation and land-use changes [45] and are being increasingly threatened by climate-driven aridification [41,46]. Sand grassland restoration has been carried out for decades, including seeding of native species [47,48,49].

These habitats are situated in the temperate region of Hungary, exhibiting sub-Mediterranean and continental climatic influences [46]. The mean annual temperature ranges between 10.5 and 11.5 °C, and total annual precipitation is 500–550 mm (https://met.hu). The soils are calcareous with a low humus content (around 1%). The grasslands have a maximum total vegetation cover of 75% and are dominated by *F. vaginata* [42].

**Figure 1:**
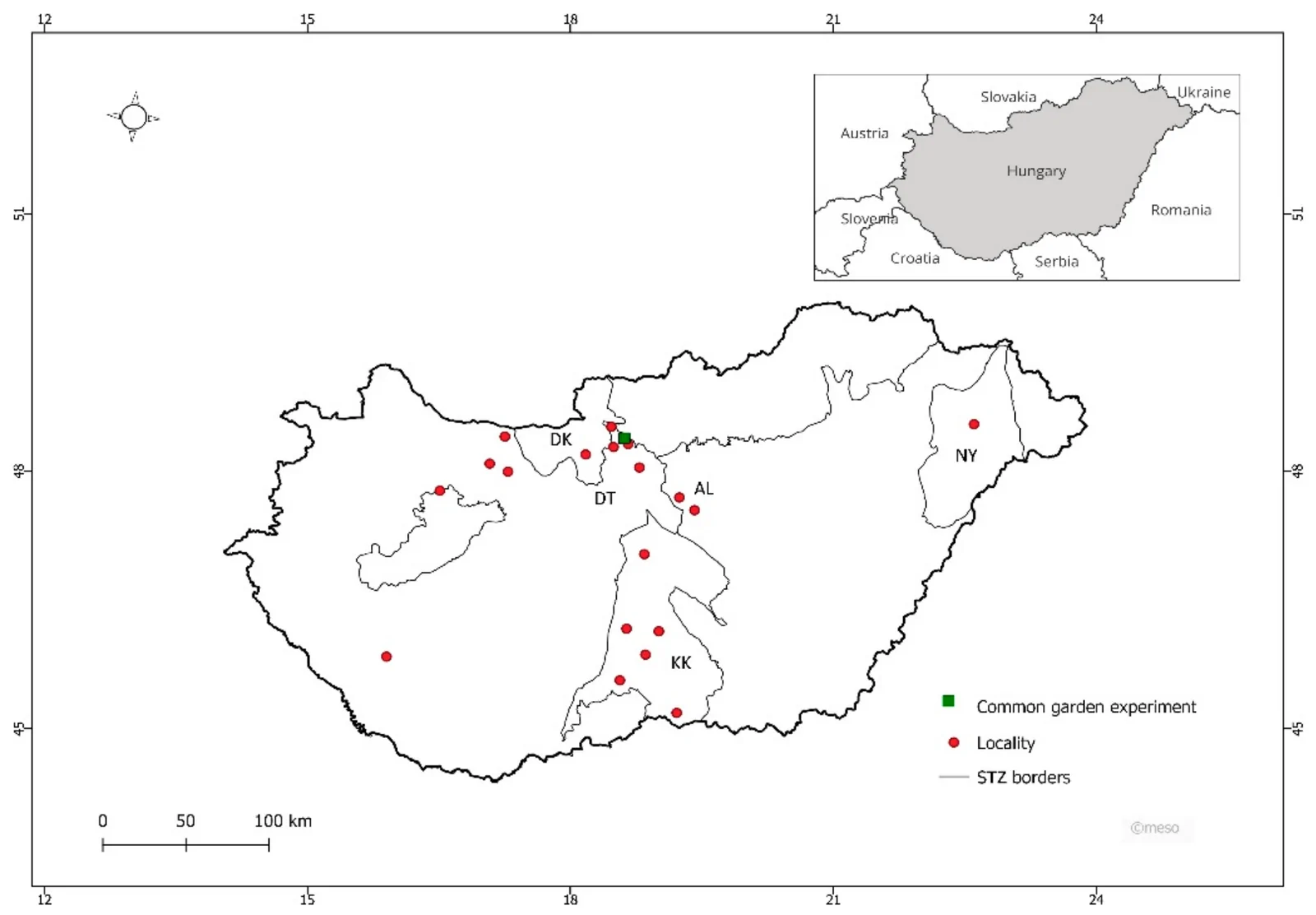
Map showing the STZs identified for Hungary by Cevallos et al. (2020) [12], the 19 sampling localities across STZs involved in this study and the geographic position of the common garden experiment set up at the National Botanical Garden in Vácrátót, Hungary. Map inset shows the position of Hungary within Europe. The STZs included in the analysis are represented with abbreviations: AL—Alföld; DK—Dunántúli-középhegység; DT—Dunántúl; KK—Kiskunság; NY—Nyírség.

The initial seed sampling area extended over 40,000 km^2^ in the Hungarian lowland. Sampling sites were selected based on the literature to represent the broad distribution of sand grassland communities across different STZs. The STZs used in this study were developed by Cevallos et al. [12] through biogeographic modelling using environmental and vegetation data, to support ecological restoration planning in Hungary. Seven STZs were identified originally, and seeds of *F. vaginata* were originally collected in 2019 from 29 localities across five STZs. The present experiment included samples from all five STZs (Figure 1).

### 2.3. Common Garden Experiments

A common garden experiment was conducted by Cevallos et al. [38] between 2019 and 2020 at the National Botanical Garden in Vácrátót, Hungary (47.708° N, 19.237° E, Figure 1) on first-generation individuals (F_0_) of four plant species. In the summer of 2021, we collected seeds of one species (*F. vaginata*) from plants of the F_0_ generation that produced a sufficient number of seeds (≥30 seeds per plant), resulting in a reduced number of localities per STZ represented in the F_1_ generation. The F_1_ generation maintained representation across all five STZs, including 19 of the 29 sampled populations. Four STZs retained 63–100% of their sampled populations, whereas the Nyír-ség (NY) STZ was represented by one of its three populations (Table S1).

**Figure 2:**
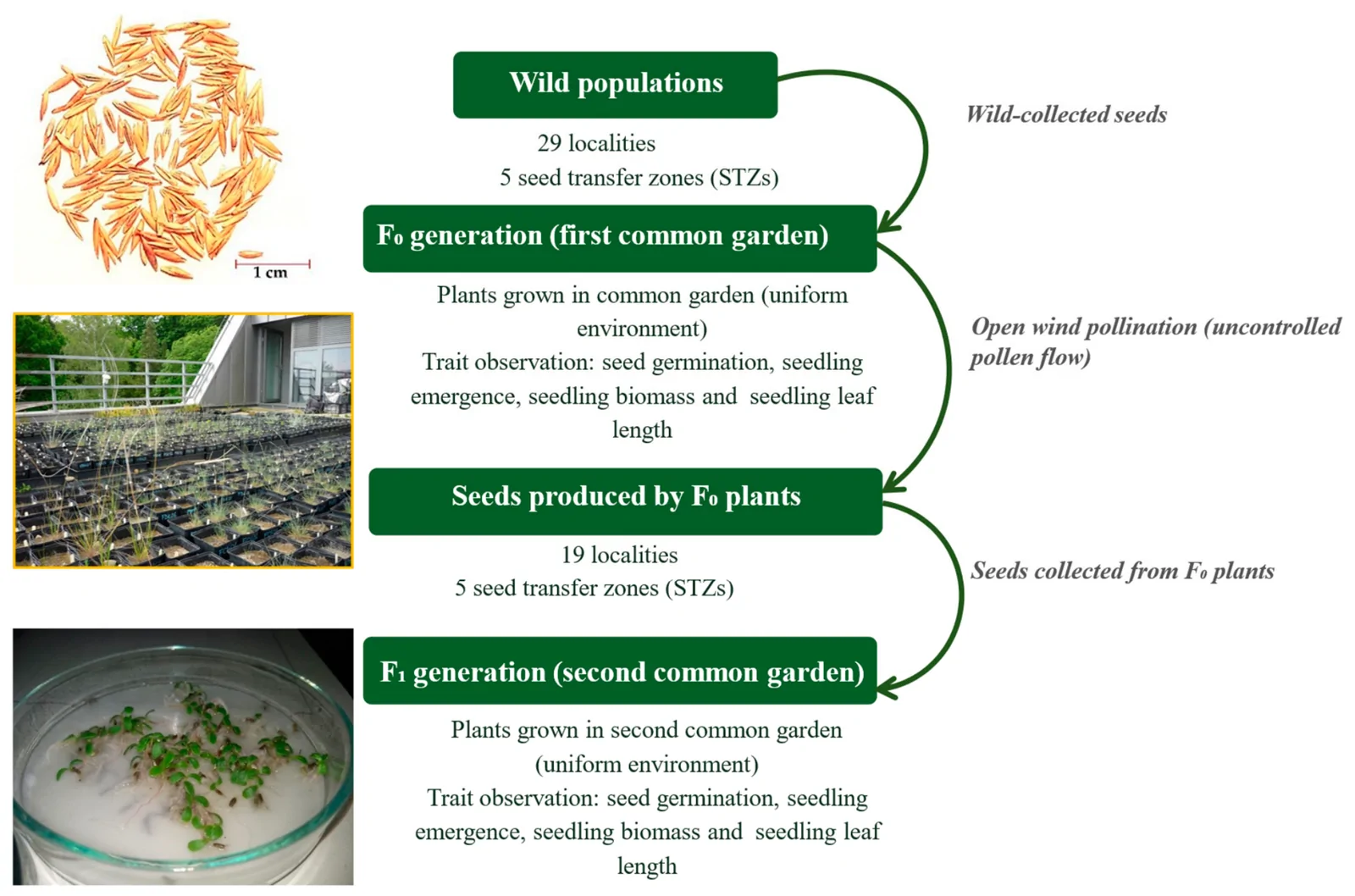
Schematic representation of the sampling design and experimental workflow. Wild-collected seeds from 29 localities across five STZs were previously used to establish the F_0_ common garden. Seeds produced by F_0_ plants under uncontrolled open wind pollination were collected from maternal plants with sufficient seed production to establish the F_1_ generation (19 localities across five STZs). F_1_ seeds were used in both a common garden pot experiment and a controlled germination experiment to quantify seed germination, seedling emergence, seedling biomass, and seedling leaf length. The photographs (from top to bottom) show seeds collected from wild populations, mature plants from the F_0_ pot experiment, and the F_1_ seed germination experiment.

We performed a second-generation (F_1_) common garden experiment between 2022 and 2023 to examine whether initial locality-level variation observed in seedling emergence, dry biomass and leaf length was still detectable. Seeds of *F. vaginata* were sown in the same common garden and following the same design used by Cevallos et al. [38]. Samples for the F_1_ experiment originated from five STZs and 19 localities, with 1–10 localities per STZ and 3–11 plants per locality (Figure 2, Table S2). Pots were filled with construction sand, and 6–15 seeds per plant were placed in a pot at a depth of 0.2–0.3 cm using a wet toothpick. Pot dryness was assessed visually by regular inspection, and watering was applied when the soil surface appeared dry. At each watering event, all pots were watered simultaneously to full soil moisture replenishment to maintain comparable moisture conditions throughout the experiment. Pot positions were randomized at the start of the experiment to minimize potential positional effects.

#### 2.3.1. Seedling Emergence, Leaf Length and Dry Biomass

We recorded seedling emergence in seeded pots monthly between November 2022 and April 2023. After recording emergence, we gently removed all seedlings except one from each pot. The aboveground parts of the uprooted seedlings were placed in paper envelopes and dried at room temperature for two weeks. Roots were not measured as the tangled roots could not be separated by specimen. The length of the longest leaf per seedling was measured with a ruler with 0.01 cm accuracy. The biomass of the dried samples was measured using a Sartorius BL 120S analytical balance (Sartorius AG, Göttingen, Germany) with 0.0001 g accuracy.

#### 2.3.2. Seed Germination Experiments in the Growth Chamber

A random subset of 3–6 maternal plants per locality of the F_0_ generation was selected for assessing seed germination capacity in vitro. We placed 35 seeds per maternal seed family in a single sterile Petri dish lined with wet filter paper (except for four Petri dishes that had only 4, 6, 17 and 19 seeds) (Table S3). Petri dishes were incubated in an Aralab climatic chamber (FITOCLIMA D1200 PLH; Aralab – Equipamentos de Laboratório e Electromecânica Geral, Lda., Rio de Mouro, Sintra, Portugal) under controlled conditions: 20 °C, 8 h light/16 h dark photoperiod, and 50% relative humidity. The germination procedure followed the protocols of Peti et al. [50] and RBGK [51]. Mould-infected or discoloured seeds were routinely removed, and the Petri dishes were rehydrated weekly or whenever the filter paper appeared dry. Germination was defined as the emergence of a radicle at least 2 mm in length. Germinated seeds were counted and removed from the Petri dishes weekly over a five-week period.

### 2.4. Statistical Analyses

We fitted two sets of models in the glmmTMB package in R [52] to examine the effects of locality and STZ on seed germination, seedling emergence, aboveground biomass and leaf length of seedlings in the F_0_ and F_1_ generations. Germination and emergence were modelled using a beta-binomial error distribution with the response specified as the number of successes and failures (accounting for variation in the initial number of seeds sowed), whereas biomass and leaf length were modelled using Gaussian error distribution. For this purpose, we used a subset of the F_0_ data from Cevallos et al. [38] to match maternal plants to the newly collected F_1_ dataset, avoiding differences arising from uneven dataset comparisons. We acknowledge that the F_1_ generation represents only the more fecund component of the original populations.

To test the effect of locality on germination and emergence, we fitted Generalized Linear Models (GLMs) with locality as the only explanatory variable. To model the response of biomass and leaf length, we fitted Generalized Linear Mixed-Effects Models (GLMMs) with locality specified as the fixed effect and pot identity as the random effect (Table S4). GLMMs were particularly useful as these models were designed to handle unbalanced designs and uneven replication across groups. We initially planned to refit the locality models to account for potential spatial autocorrelation in the residuals. However, the Moran’s I test performed using the moran package in R [53] did not detect any significant spatial autocorrelation for either response variable, making the correction unnecessary.

To verify the results by Cevallos et al. [38] with our model structure and the reduced dataset, we also tested the effects of STZ on the measured traits, fitteing GLMMs. For the germination and emergence models, STZ was specified as the fixed effect and locality as the random effect. For biomass and leaf length models, pot identity was nested within locality as a random effect to avoid pseudoreplication and account for multiple plants collected from each pot (Table S4).

We used model estimates to compare the effect sizes of locality and STZ on each measured trait between the F_0_ and F_1_ generations. In both the locality and STZ models, we assessed model performance across generations using the Mean Squared Error (MSE), which reflects average prediction error, and conditional R^2^, which quantifies the proportion of variance explained by both the fixed and random effects. The statistical significance of the fixed effects was assessed using likelihood ratio tests (LRTs), and the reported *p*-values were obtained by comparing models with and without the fixed effect of interest. We report estimated marginal means (EMMs) and corresponding 95% confidence intervals for seed germination, seedling emergence, seedling biomass, and seedling leaf length calculated from the fitted models (Table S5).

To explore how much variation attributable to locality was shared with STZ and how much was exclusive due to locality alone, we performed a complementary variance partitioning analysis using a subset of the data, limited to two STZs (DT and KK, with 10 and 5 localities, respectively), as these were the only STZs with more than three localities, which is necessary for a meaningful comparison. We first fitted Generalized Linear Models for seed emergence and seedling germination (GLMs) and Linear Mixed-Effects Models (LMMs) for seedling biomass and seedling leaf length (Table S6). In these models, STZ and locality were treated as fixed explanatory variables because the aim was to partition their respective contributions to trait variation. For the biomass and leaf length models, pot identity was included as a random effect to account for pseudoreplication. The analyses were conducted at the pot level; consequently, localities contributed proportionally to the number of sampled maternal plants (3–9 pots per locality), to the precision with which their mean response was estimated. We extracted the R^2^ measures representing the explanatory power of models including STZ, locality, or both. For the GLMs, the explanatory power was quantified using deviance-based pseudo-R^2^, whereas for the mixed-effects models, it was quantified using Nakagawa’s marginal R^2^. We then conducted the variance partitioning analysis to quantify the unique and shared contributions of STZ and locality to each response trait according to the formulas: the unique contribution of STZ = R^2^(STZ + Locality) − R^2^(Locality); the unique contribution of locality = R^2^(STZ + Locality) − R^2^(STZ); and the shared contribution = R^2^(STZ) + R^2^(Locality) − R^2^(STZ + Locality). In all models, biomass and leaf length were log-transformed to meet model assumptions.

## 3. Results

Locality had a significant effect on all measured traits in the F_0_ generation (*p* < 0.01; Table 1). In the F_1_ generation, locality did not significantly influence germination or emergence (*p* > 0.05), but it had a significant effect on seedling biomass and leaf length (*p* < 0.05; Table 1).

**Figure 3:**
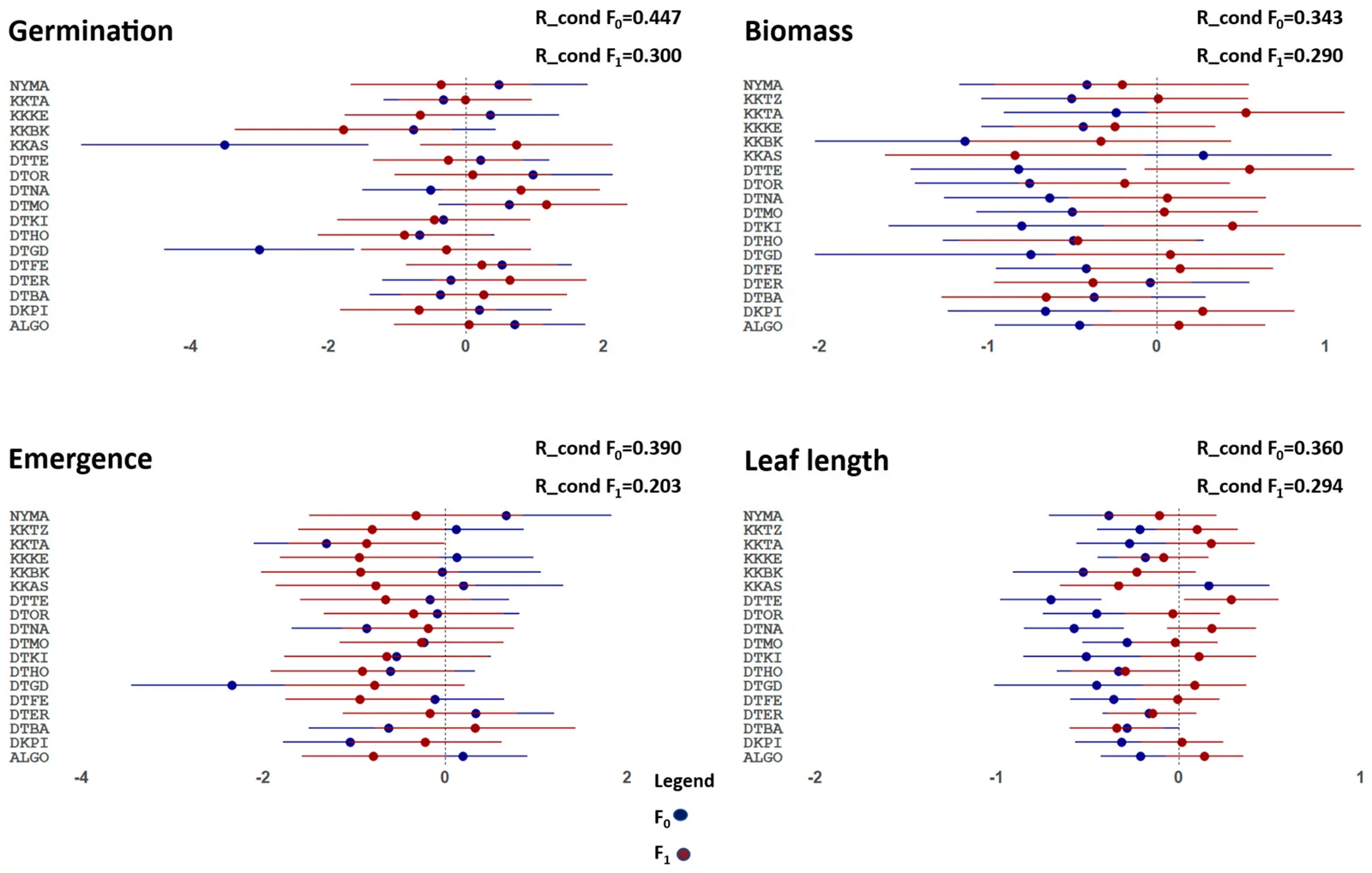
Coefficient plots comparing the effect size (slope and 95% confidence intervals) of localities and model conditional R^2^ values in the F_0_ (blue) and F_1_ (red) generations, derived from models of seed germination, seedling biomass, seedling emergence, and seedling leaf length of *Festuca vaginata*. Positive slope values indicate a positive effect of the respective locality on each response variable relative to the reference locality, whereas negative values indicate a negative effect. The first two letters of each locality code denote the STZ (see Figure 1 for STZ names). Localities are: Magy (NYMA), Tázlár (KKTZ), Táborfalva (KKTA), Kéleshalom (KKKE), Bugac (KKBK), Ásotthalom (KKAS), Tece (DTTE), Oroszlány (DTOR), Nagybajom (DTNA), Mocsa (DTMO), Kisoroszi (DTKI), Horány (DTHO), Gödöllő (DTGD), Fenyőfő (DTFE), Erdőkertes (DTER), Bársonyos (DTBA), Pilisvörösvár–Kálváriadomb (DKPI), and Göbölyjárás (ALGO).

The magnitude and variability of individual locality effects differed among traits and between generations. For germination and emergence, effect sizes tended to be more variable in the F_0_ generation, with several more extreme locality effects. In contrast, for seedling biomass and leaf length, locality effects tended to be closer to the reference in F1, particularly for leaf length (Figure 3). Additionally, several localities exhibited shifts in the direction of their effects between generations, switching from negative to positive (and vice versa) relative to the reference site (Figure 3). No significant spatial autocorrelation was detected in any locality model in either generation (Moran’s I test, *p* > 0.05; Table 1).

STZ did not have a significant effect on any of the measured traits (Table 2). 

**Figure 4:**
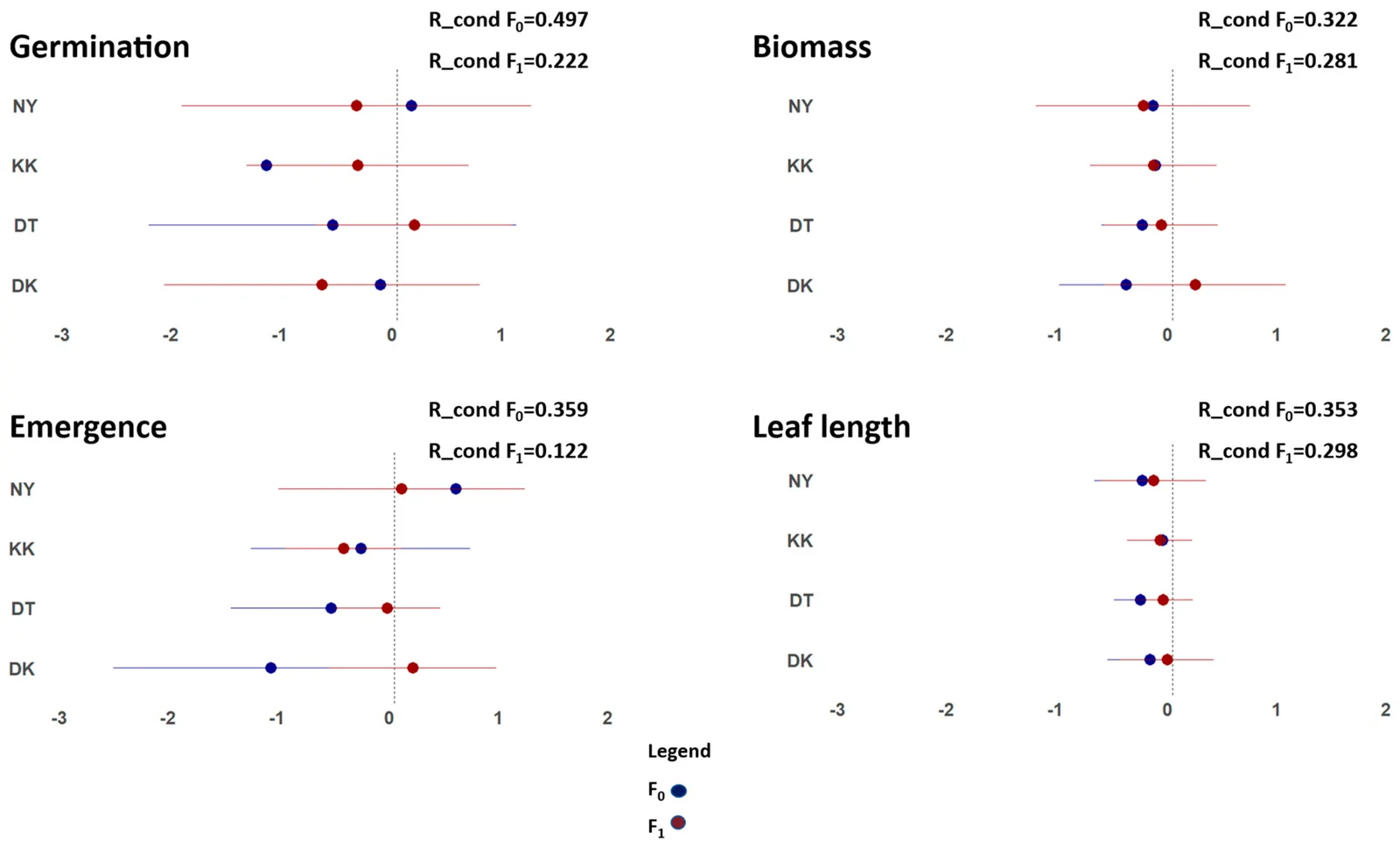
Coefficient plots comparing the effect size (slope and 95% confidence intervals) of STZs and model conditional R^2^ values in the F_0_ (blue) and F_1_ (red) generations derived from models of seed germination, seedling biomass, seedling emergence, and seedling leaf length of *Festuca vaginata*. For STZ names, see Figure 1. Positive slope values indicate a positive effect of the respective STZ on each response variable relative to the reference STZ and negative values indicate a negative effect.

Similarly, individual STZ effects were generally weaker in F_1_ than in F_0_, with only few exceptions (Figure 4).

Conditional R^2^ values were consistently lower in F_1_ than in F_0_ in both the locality and STZ models. In the STZ models, the MSE values were higher in the F_1_ models except for emergence, whereas in the locality models, the MSE was higher in F_0_ for seed germination and seedling emergence but higher in F_1_ for seedling biomass and leaf length (Table 1 and Table 2).

**Figure 5:**
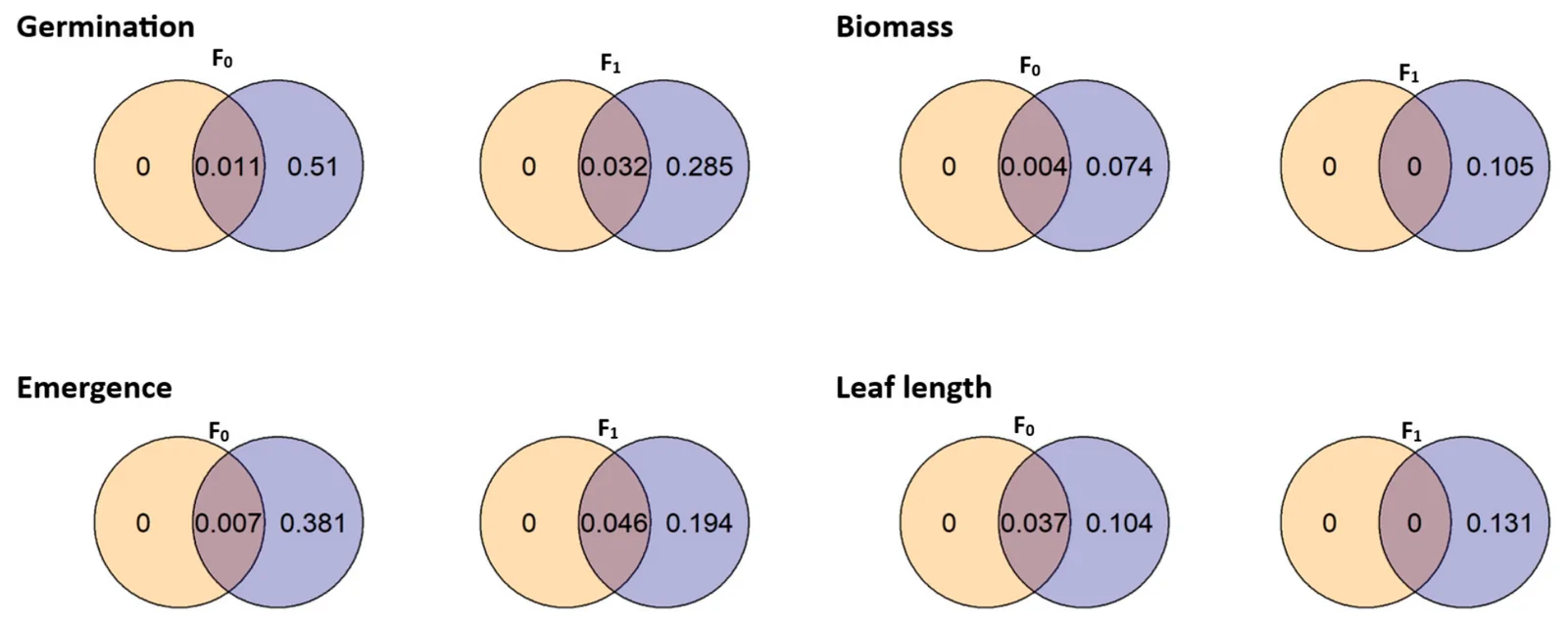
Venn diagrams showing the relative explanatory power of STZ (orange) and locality (purple) for seed germination, seedling emergence, seedling biomass and seedling leaf length of *Festuca vaginata* in the F_0_ and F_1_ generations. Analyses were restricted to the two STZs with sufficient locality replication: Dunántúl (10 localities) and Kiskunság (5 localities). For seedling emergence and germination, explanatory power was quantified using deviance-based pseudo-R^2^ derived from Generalized Linear Models (GLMs). For seedling biomass and leaf length, explanatory power was quantified using Nakagawa’s marginal R^2^ derived from Linear Mixed-Effects Models, representing the explanatory power of the fixed effects. The diagrams compare models including STZ, locality, or both as fixed effects.

In the complementary variance partitioning analysis based on two STZs, locality consistently explained substantially more variation than STZ when considered independently, whereas models including both predictors did not explain more variation than locality alone (Figure 5, Table S7).

## 4. Discussion

We investigated whether locality-level variation in seed germination and seedling traits of a wind-pollinated perennial grass observed in the first generation of a common garden experiment persisted in offspring despite uncontrolled gene flow. Our results align with the previous analyses by Cevallos et al. [38] that suggested weak regional differentiation in *F. vaginata*, supporting the idea that provenance rules based on broad regional zones may be less critical than accounting for determinants of variation in local population performance. Importantly, our findings highlight that the transgenerational stability of population differentiation may be trait-specific and require further detailed investigations.

As expected, the magnitude of trait variation at the locality level was lower in the second generation. While we hypothesize that rapid environmental homogenization or maternal effects may be responsible, we must also consider that genetic mixing due to uncontrolled gene flow or selection bias due to high-fecundity individuals involved in the study may have reduced the phenotypic variation (see further details below). However, the strength of the homogenizing effect depended on the trait: differences between localities in germination capacity and seedling emergence disappeared in the F_1_ generation, but they were detectable in both generations for seedling biomass and leaf length. Due to the experimental set up, our experiment cannot ascertain a clear cause of the disappearance of locality effects on seed germination and seedling emergence. However, evidence suggests that maternal effects are stronger for traits under strong selection [54] and with limited phenotypic plasticity; this may apply to early life stages such as seed germination and seedling emergence, where opportunities for post-dispersal adjustment are constrained [22]. In harsh, resource-poor environments like dry sand grasslands, seeds germinate under narrow moisture and temperature windows, as unreliable cues would increase the risk of seedling mortality. This can constrain the plasticity of germination timing and, consequently, germination strategies often combine plastic responses with bet-hedging mechanisms depending on environmental predictability [55,56]. Maternal provisioning may therefore be an important mechanism for matching offspring phenotype to the environment, forming part of a conservative strategy typical of species in dry sand grasslands [57,58].

In contrast, the persistence of variation in seedling biomass and leaf length suggests a more stable signature of origin that appears to outlast the homogenizing effects of the common garden. Seedling trait variation was concentrated at the locality level, and it was not driven by the geographic distance between populations. Fitness at restored sites is generally expected to decline as the geographical and ecological distance from the source population increases because of simultaneous environmental divergence and genetic isolation [11,23,30]. However, the dependence of differentiation between populations based on simple geographic distance is not consistently supported by empirical evidence, and patterns may be system dependent [59]. In wind-pollinated species, spatial genetic structuring can be particularly weak over large geographic distances [60]. In *F. vaginata*, we expected environmental variation to act as a barrier to gene flow and promote adaptive divergence between STZs and populations [61,62,63]. The two STZs with sufficient locations for variance partitioning, DT (Dunántúl) and KK (Kiskunság), represented biogeographically distinct regions, separated by a large area by the Danube River. KK was characterized by extensive inland sand dunes, while sandy areas in the DT region were more fragmented and the region experiences a milder climate [64]. Despite these environmental differences, the variance partitioning limited to these two STZs showed that models including both STZ and locality did not explain more variation than locality alone. Similar inconsistencies between STZ and phenotypic differentiation have been reported in other common garden studies, suggesting that broad regional classifications do not necessarily capture biologically meaningful patterns of adaptive variation [65].

These finding align with results from other common garden studies showing that strong regional differentiation is not always observed even across environmentally distinct areas [18,23]. Instead, the persistence of population differentiation in the F1 generation despite the potential for pollen-mediated gene flow strengthens the argument for a site-specific genetic basis for vegetative trait variation, which could be maintained by a number of factors. For example, biomass and leaf traits could be governed by polygenic architecture, with traits being controlled by many loci, and thus recombination cannot homogenize the population in a single generation [66,67]. In wind-pollinated plants, effective pollen dispersal does not necessarily result in effective gene flow as successful fertilization depends on multiple ecological and biological filters, including phenological mismatches, spatial structure, pollen competition, and compatibility systems that can limit mating [39]. However, because we did not control for paternity, we cannot definitively disentangle the effects of genetic recombination from other transgenerational factors.

The observed sign reversals in location effects across generations point to a reordering of relative performance among sites, consistent with genotype-by-environment interactions producing transgenerational shifts in relative performance [56]. However, many of these shifts were associated with substantial uncertainty and should therefore be interpreted cautiously.

High trait variance among provenances is commonly observed across herbaceous species [32,61,68], and this variation can be both plastic and genetically determined [69]. A genetic basis of variation in seedling and juvenile biomass was also suggested by a common garden study of seven herbaceous species, which did not detect any maternal environmental effects on offspring performance [70], while another study reported maternal effects on seedling height at two provenances [71]. This mixed evidence suggests that the genetic control of these traits may be species or life-history specific, requiring careful investigation.

### 4.1. Applications in Ecological Restoration

How do these findings inform seed sourcing for ecological restoration? Although restoration practitioners often prefer local seeds, such sources are frequently unavailable in the necessary quantities or with the necessary genetic diversity [5,72,73]. Meeting restoration goals will require a balance between strict local provenance and more flexible sourcing approaches [34]. As we found weak regional patterns in trait variability, our results suggest that a more flexible provenancing approach may be viable for *F*. *vaginata*. This view is reinforced by empirical studies showing that regional genetic differentiation is often weak in wind-pollinated species, likely due to high levels of gene flow [18,23]. From a management perspective, post-seeding management practices, rather than seed origin, often have a stronger influence on restoration success [74], and using mixed provenances can improve the survival of reintroduced, protected species [75]. On the other hand, our results also suggest that incorporating locality-level data into restoration is necessary in wind-pollinated species. If differences between germination and seedling trait responses to the common new environment persist, there are demographic consequences for the novel population. Even if the initial establishment is successful due to homogeneous germination and seedling emergence responses, the performance and persistence of individuals may still be dictated by the site-specific signature of origin, creating a complex demographic mosaic at the restoration site. This novel admixture could have contrasting outcomes: positive outcomes if it has adaptive advantages in novel or changing environments [76], or negative outcomes if it has an increased risk of maladaptation at the restored sites, losing the evolutionary potential to adapt to future environmental changes [29,77]. The persistence of local-scale variation in early vegetative traits suggests that further research is needed to guide site-specific decisions, bearing in mind that morphological differences among populations are meaningful only if they have a genetic basis [78].

### 4.2. Limitations and Future Prospects

Because in our study the F_1_ generation could only be established from F_0_ plants producing sufficient seed, it represented a non-random subset of the original population. This may have reduced the representation of low-fecundity genotypes, and therefore part of the reduction in trait variation between generations could reflect both this selection process and the more homogeneous experimental environment. However, the selected populations retained representation across all geographic regions. Furthermore, analyses of the complete F_0_ dataset for additional adult traits (unpublished, under review) similarly found only weak associations between STZ and phenotypic variation. These findings are consistent with the published analysis of seed traits by Cevallos et al. [38], which also found limited evidence that geographic distance or STZ explained trait differences. Together, these independent analyses suggest that the sampling constraint was unlikely to have affected the main conclusions.

Due to uncontrolled gene flow between individuals in the first common garden experiment, we could not distinguish the relative contributions of pollen-mediated gene flow, phenotypic plasticity, maternal environmental effects, and other environmentally determined differences to the observed changes in seed germination and seedling emergence between generations, which is a limitation of open-pollinated common garden studies of outcrossing perennial species [79]. As a wind-pollinated Poaceae species, *F. vaginata* is likely to experience pollen-mediated gene flow in common garden settings, particularly because gene flow in wind-pollinated grasses is greatest among nearby individuals and decreases with distance [80]. Consequently, the reduced locality effects observed in the F_1_ generation could be partially explained by pollen-mediated gene flow and increased genetic admixture, processes that can reduce population differentiation over generations [77,81]. Future research combining multi-generation common garden experiments with controlled pollination and molecular marker or parentage analyses would enable the quantification of pollen-mediated gene flow and help disentangle its contribution from maternal environmental effects and phenotypic plasticity, thereby improving our understanding of STZs and locality effects and informing seed sourcing strategies for *F. vaginata* and other wind-pollinated grasses [77]. Nevertheless, because our common garden mimicked the genetic mixing inherent in restoration projects, it provides a valuable (yet mechanistically ambiguous) preview of the possible demographic outcomes arising when different provenances are pooled.

Caution is also warranted in generalizing results from one or few species to guide conservation practice [16,24]. However, our results suggest that *F. vaginata* may be a good model species to develop provenance guidelines for translocations of wind-pollinated species in sand grasslands.

## 5. Conclusions

Our results suggest that transgenerational stability under shared environmental conditions may be trait-dependent, with traits related to seed germination responding differently from vegetative traits. Therefore, shared cultivation settings in *F. vaginata* may create novel demographic pathways where uniform germination may ensure successful initial establishment, whereas a persistent signature of origin in vegetative traits carries both potential benefits (genetic admixture) and risks (loss of species’ functional identity). Testing whether this pattern is consistent across species and environments represents an important direction for future research and could improve our understanding of how common garden cultivation influences transgenerational phenotypic variation and its implications on ecological restoration.

Although our results indicate that provenance guidelines based on broadly defined STZs may warrant re-evaluation for *F. vaginata*, this interpretation should be viewed in light of the study’s limitations, including the focus on a single wind-pollinated species, early life-stage traits, one common garden environment with uncontrolled gene flow, and the absence of genetic data. The patterns detected here may serve as a baseline for future studies combining reciprocal transplant experiments with genomic analyses across multiple generations and life stages, offering more powerful tools for determining whether the observed phenotypic patterns reflect phenotypic plasticity, genetic differentiation, or their interaction.

## Figures and Tables

**Table 1:** Summary of model estimates and performance metrics for models of locality effects on emergence, germination, biomass and leaf length of *Festuca vaginata* in the F_0_ and F_1_ generations. β (estimate) represents the effect size of each trait, SE (β) is the standard error associated with the estimate, MSE is the Mean Squared Error of the model’s predictions, R2_cond is the proportion of variance explained by the model, *p*-value is the statistical significance of the estimates, and Moran’s I *p*-value is the significance of the test for spatial autocorrelation in model residuals.

Trait	Generation	β	SE (β)	MSE	R2_Cond	*p*-Value	Moran’s I *p*-Value
Seed germination	F_0_	0.519	0.384	0.096	0.447	**<0.001**	0.712
	F_1_	0.367	0.405	0.055	0.300	0.1456	0.308
Seedling emergence	F_0_	0.676	0.255	0.034	0.390	**<0.001**	0.593
	F_1_	1.620	0.297	0.025	0.203	0.100	0.415
Seedling biomass	F_0_	−4.048	0.154	0.453	0.343	**0.010**	0.910
	F_1_	−3.976	0.163	0.721	0.290	**0.009**	0.989
Seedling leaf length	F_0_	1.789	0.080	0.129	0.360	**<0.001**	0.979
	F_1_	1.344	0.074	0.157	0.294	<**0.001**	0.421

Bold *p*-values indicate statistically significant results (*p* < 0.05).

**Table 2:** Summary for model estimates and performance metrics for models of seed transfer zone (STZ) effects on seed germination, seedling emergence, seedling biomass and seedling leaf length of *Festuca vaginata* in the F_0_ and F_1_ generations. β (estimate) represents the effect size of each trait, SE (β) is the standard error associated with the estimate, MSE is the Mean Squared Error of the model’s predictions, R2_cond is the proportion of variance explained by the model and *p*-value is the statistical significance of the estimates.

Trait	Generation	β	SE (β)	MSE	R^2^_Cond	*p*-Value
Seed germination	F_0_	0.883	0.757	0.037	0.497	0.566
	F_1_	0.385	0.398	0.054	0.222	0.333
Seedling emergence	F_0_	0.768	0.421	0.036	0.359	0.198
	F_1_	1.210	0.255	0.028	0.122	0.382
Seedling biomass	F_0_	−4.276	0.174	0.455	0.322	0.258
	F_1_	−3.901	0.222	0.722	0.281	0.775
Seedling leaf length	F_0_	1.683	0.107	0.130	0.353	0.062
	F_1_	1.414	0.125	0.157	0.298	1.000

## Data Availability

The original contributions presented in this study are included in the article/Supplementary Material. Further inquiries can be directed to the corresponding authors.

## Supplementary Materials

The following supporting information can be downloaded at: https://www.mdpi.com/article/10.3390/plants15172556/s1, Table S1. Number of localities sampled in the F_0_ experiment and retained in the F_1_ experiment for each seed transfer zone (STZ); Table S2. Number of maternal seed families and the total, minimum, and maximum numbers of seeds sown and seedlings emerged in the common garden pot experiment for each locality and seed transfer zone (STZ). Maternal seed families represent the number of maternal plants contributing seeds to the common garden experiment, with each maternal family represented by a single pot; Table S3. Number of maternal seed families and the total, minimum, and maximum numbers of seeds sown and seeds germinated in the germination experiment (in the germination chamber) for each locality and seed transfer zone (STZ). Maternal seed families represent the number of maternal plants contributing seeds to the germination experiment, with each maternal family represented by a single Petri dish; Table S4. Structure of the generalized linear models (GLMs) and generalized linear mixed models (GLMMs) used to analyse seed germination, seedling emergence, biomass, and leaf length. Germination and emergence were modelled as the numbers of successes and failures using beta-binomial GLMs and GLMMs. Biomass and leaf length were analysed using Gaussian GLMMs fitted to log-transformed response variables. All models were fitted using the glmmTMB package in R (Brooks et al., 2017) [52]; Table S5. Estimated marginal means (EMMs) and 95% confidence intervals for germination percentage, emergence percentage, biomass, and leaf length for each seed transfer zone (STZ) in the F_0_ and F_1_ generations, based on the fitted generalized linear mixed-effects models (GLMMs). Germination and emergence percentages were estimated from beta-binomial GLMMs, whereas biomass and leaf length were estimated from Gaussian GLMMs. Biomass and leaf length values are presented on the original scale after back-transformation from the log-transformed Gaussian GLMMs; Table S6. Model structures used for variance partitioning. For each response variable, three models were fitted: a model including only seed transfer zone (STZ), a model including only locality, and a model including both predictors. Explanatory power was quantified using Efron’s pseudo-R^2^ for germination and emergence models and Nakagawa’s marginal R^2^ for biomass and leaf-length mixed-effects models. All models were fitted using the glmmTMB package in R (Brooks et al., 2017) [52]; Table S7. Comparison of the explanatory power of seed transfer zone (STZ) and locality models for seed germination, seedling emergence, seedling biomass, and seedling leaf length in the F_0_ and F_1_ generations. Analyses were restricted to the two STZs with more than three localities (Dunántúl, *n* = 10 localities; Kiskunság, *n* = 5 localities). For seed germination and seedling emergence, explanatory power is presented as deviance-based pseudo-R^2^ derived from generalized linear models. For seedling biomass and seedling leaf length, explanatory power is presented as Nakagawa’s marginal R^2^ derived from linear mixed-effects models. The table compares models including seed transfer zone, locality, or both predictors.
