# Supplementary material for: Trait-Specific Patterns of Phenotypic Differentiation Among Populations After Two Generations of Common-Garden Cultivation in a Wind-Pollinated Grass
Source: Plants (Basel). 2026 Aug 22;15(17):2556. doi: 10.3390/plants15172556 (PMC13567639; doi:10.3390/plants15172556)
Supplement: Supplementary file 1 [file plants-15-02556-s001.zip › plants-4417559-supplementary.pdf]

Supporting Material

Table S1. Number of localities sampled in the F_0 experiment and retained in the F_1 experiment for each seed transfer zone (STZ).

STZ	Localities in F_0	Localities selected for F_1	F_1 % of total localities
	29	19	66
Alföld (AL)	2	2	100
Dunántúli-középhegység (DK)	1	1	100
Nyírség (NY)	3	1	33
Dunántúl (DT)	16	10	63
Kiskunság (KK)	7	5	71

Table S2. Number of maternal seed families and the total, minimum, and maximum numbers of seeds sown and seedlings emerged in the common garden pot experiment for each locality and seed transfer zone (STZ). Maternal seed families represent the number of maternal plants contributing seeds to the common garden experiment, with each maternal family represented by a single pot.

STZ	Locality	Maternal seed families (n)	Seeds sowed	Minimum seeds/pot	Maximum seeds/pot	Seedlings emerged	Minimum emerged/pot	Maximum emerged/pot	Percentage emergence (%)
Alföld (AL)	Gicei-hegy	11	165	15	15	140	11	15	84.85
	Göbölyjárás	11	150	6	15	103	3	15	68.67
Dunántúli-középhegység (DK)	Pilisvörösvár - Kálváriadomb	9	135	15	15	91	1	14	67.41
Dunántúl (DT)	Bársonyos	5	75	15	15	67	12	15	89.33
	Erdőkertes	6	90	15	15	74	11	15	82.22
	Fenyőfő	8	120	15	15	79	6	14	65.83
	Gödöllő	4	60	15	15	43	10	12	71.67
	Horány	4	51	6	15	35	4	11	68.63
	Kisoroszi	3	45	15	15	33	8	13	73.33
	Mocsa	7	105	15	15	84	9	14	80
	Nagybajom	6	90	15	15	74	10	14	82.22
	Oroszlány	5	75	15	15	59	9	14	78.67
	Tece	5	75	15	15	63	11	15	84
Kiskunság (KK)	Bugac	3	45	15	15	30	7	13	66.67
	Kéleshalom	6	90	15	15	60	5	13	66.67
	Táborfalva	7	105	15	15	86	10	15	81.9
	Tázlár	9	135	15	15	87	5	13	64.44
	Ásotthalom	3	45	15	15	32	9	13	71.11
Nyírség (NY)	Magy	3	45	15	15	36	11	14	80

Table S3. Number of maternal seed families and the total, minimum, and maximum numbers of seeds sown and seeds germinated in the germination experiment (in the germination chamber) for each locality and seed transfer zone (STZ). Maternal seed families represent the number of maternal plants contributing seeds to the germination experiment, with each maternal family represented by a single Petri dish.

STZ	Locality	Maternal seed families (n)	Seeds sowed	Minimum seeds/Petri dish	Maximum seeds/Petri dish	Seeds germinated	Minimum germinated/Petri dish	Maximum germinated/ Petri dish	Percentage germination (%)
Alföld (AL)	Gicei-hegy	4	140	35	35	102	16	32	72.86
	Göbölyjárás	6	210	35	35	130	11	30	61.9
Dunántúli-középhegység (DK)	Pilisvörösvár - Kálváriadomb	5	175	35	35	71	9	22	40.57
Dunántúl (DT)	Bársonyos	5	125	17	35	81	3	32	64.8
	Erdőkertes	6	210	35	35	167	24	31	79.52
	Fenyőfő	6	210	35	35	137	7	32	65.24
	Gödöllő	6	190	19	35	96	1	29	50.53
	Horány	4	109	4	35	40	1	21	36.7
	Kisoroszi	3	105	35	35	50	1	34	47.62
	Mocsa (MO)	6	210	35	35	181	27	35	86.19
	Nagybajom	6	210	35	35	163	21	35	77.62
	Oroszlány	5	175	35	35	113	18	26	64.57
	Tece	5	210	35	35	107	11	33	50.95
Kiskunság (KK)	Bugac	3	76	6	35	17	0	14	22.37
	Kéleshalom	6	210	35	35	95	1	24	45.24
	Táborfalva	6	210	35	35	122	3	35	58.1
	Tázlár	5	175	35	35	103	5	26	58.86
	Ásotthalom	3	105	35	35	80	21	34	76.19
Nyírség (NY)	Magy	3	105	35	35	53	15	21	50.48

Table S4. Structure of the generalized linear models (GLMs) and generalized linear mixed models (GLMMs) used to analyse seed germination, seedling emergence, biomass, and leaf length. Germination and emergence were modelled as the numbers of successes and failures using beta-binomial GLMs and GLMMs. Biomass and leaf length were analysed using Gaussian GLMMs fitted to log-transformed response variables. All models were fitted using the glmmTMB package in R (Brooks et al., 2017).

Response variable	Model formula	Model type	Distribution
**Locality models**			
Germination	cbind (Germinated seeds, Non-germinated seeds) ~ Locality	GLM	Beta-binomial
Emergence	cbind (Emerged seedlings, Non-emerged seeds) ~ Locality	GLM	Beta-binomial
Biomass	log (Biomass) ~ Locality + (1 | pot id)	GLMM	Gaussian
Leaf length	log (Leaf length) ~ Locality + (1 | pot id)	GLMM	Gaussian
**Seed transfer zone (STZ) models**			
Germination	cbind (Germinated seeds, Non-germinated seeds) ~ STZ + (1 | Locality)	GLMM	Beta-binomial
Emergence	cbind (Emerged seedlings, Non-emerged seeds) ~ STZ + (1 | Locality)	GLMM	Beta-binomial
Biomass	log (Biomass) ~ STZ + (1 | Locality/pot id)	GLMM	Gaussian
Leaf length	log (Leaf length) ~ STZ + (1 | Locality/ pot id)	GLMM	Gaussian

Table S5. Estimated marginal means (EMMs) and 95% confidence intervals for germination percentage, emergence percentage, biomass, and leaf length for each seed transfer zone (STZ) in the F_0 and F_1 generations, based on the fitted generalized linear mixed-effects models (GLMMs). Germination and emergence percentages were estimated from beta-binomial GLMMs, whereas biomass and leaf length were estimated from Gaussian GLMMs. Biomass and leaf length values are presented on the original scale after back-transformation from the log-transformed Gaussian GLMMs.

**Germination (%)**				
**STZ**	**F_0 Germination (%)**	**95% CI (F_0)**	**F_1 Germination (%)**	**95% CI (F_1)**
Alföld (AL)	70.7	35.4–91.4	67.7	46.2-83.7
Dunántúli-középhegység (DK)	67.5	20.0–94.5	42.6	18.4-70.9
Dunántúl (DT)	57.2	40.6–72.4	61.6	52.2-70.2
Kiskunság (KK)	45.2	23.8–68.4	51.8	38.1-65.3
Nyírség (NY)	73.4	22.5–96.3	50.4	20.4-80.1
**Emergence (%)**				
**STZ**	**F_0 Emergence (%)**	**95% CI (F_0)**	**F_1 Emergence (%)**	**95% CI (F_1)**
Alföld (AL)	68.3	48.6–83.1	77.0	67.0–84.7
Dunántúli-középhegység (DK)	41.0	17.5–69.4	68.0	51.1–81.2
Dunántúl (DT)	54.6	44.6–64.3	77.0	71.6–81.7
Kiskunság (KK)	61.3	47.1–73.8	70.1	61.7–77.3
Nyírség (NY)	79.1	46.1-94.4	78.4	54.4-91.7
**Biomass (g)**				

STZ	F_0 Biomass	95% CI (F_0)	F_1 Biomass	95% CI (F_1)
Alföld (AL)	0.014	0.010–0.020	0.020	0.013–0.031
Dunántúli-középhegység (DK)	0.009	0.005–0.016	0.023	0.012–0.044
Dunántúl (DT)	0.010	0.008–0.012	0.018	0.014–0.022
Kiskunság (KK)	0.013	0.010–0.017	0.017	0.012–0.023
Nyírség (NY)	0.012	0.006-0.023	0.015	0.007-0.033

Leaf length (cm)

Alföld (AL)	F_0 Leaf length	95% CI (F_0)	F_1 Leaf length	95% CI (F_1)
Dunántúli-középhegység (DK)	5.381	4.360–6.640	4.111	3.224–5.241
Dunántúl (DT)	4.361	3.161–6.018	3.905	2.740–5.566
Kiskunság (KK)	4.002	3.574–4.481	3.772	3.348–4.251
Nyírség (NY)	4.914	4.193–5.759	3.655	3.076–4.343
Alföld (AL)	4.073	2.772–5.984	3.445	2.272–5.223

Table S6. Model structures used for variance partitioning. For each response variable, three models were fitted: a model including only seed transfer zone (STZ), a model including only locality, and a model including both predictors. Explanatory power was quantified using Efron's pseudo-R^2 for germination and emergence models and Nakagawa's marginal R^2 for biomass and leaf-length mixed-effects models. All models were fitted using the glmmTMB package in R (Brooks et al., 2017)

**Response variable**	**STZ model**	**Locality model**	**Full model**	**Model type**	**Measure of explanatory power**
Germination	cbind (Germinated seeds, Non-germinated seeds) ~ STZ	cbind (Germinated seeds, Non-germinated seeds) ~ Locality	cbind (Germinated seeds, Non-germinated seeds) ~ STZ + Locality	GLM	Efron's pseudo-R^2
Emergence	cbind (Emerged seedlings, Non-emerged seeds) ~ STZ	cbind (Emerged seedlings, Non-emerged seeds) ~ Locality	cbind (Emerged seedlings, Non-emerged seeds) ~ STZ + Locality	GLM	Efron's pseudo-R^2
Biomass	log (Biomass) ~ STZ + (1 | Pot id)	log (Biomass) ~ Locality + (1 | Pot id)	log (Biomass) ~ STZ + Locality + (1 | Pot id)	GLMM	Nakagawa's marginal R^2
Leaf length	log (Leaf length) ~ STZ + (1 | Pot id)	log (Leaf length) ~ Locality + (1 | Pot id)	log (Leaf length) ~ STZ + Locality + (1 | Pot id)	GLMM	Nakagawa's marginal R^2

Table S7. Comparison of the explanatory power of seed transfer zone (STZ) and locality models for seed germination, seedling emergence, seedling biomass, and seedling leaf length in the F_0 and F_1 generations. Analyses were restricted to the two STZs with more than three localities (Dunántúl, *n* = 10 localities; Kiskunság, *n* = 5 localities). For seed germination and seedling emergence, explanatory power is presented as deviance-based pseudo-R^2 derived from generalized linear models. For seedling biomass and seedling leaf length, explanatory power is presented as Nakagawa's marginal R^2 derived from linear mixed-effects models. The table compares models including seed transfer zone, locality, or both predictors.

Trait	Generation	STZ R^2 (%)	Locality R^2 (%)	STZ+ locality (%)
Seed germination	F_0	1.1	52.1	52.1
	F_1	3.2	31.7	31.7
Seedling emergence	F_0	0.7	38.7	38.7
	F_1	4.6	24	24
Seedling biomass	F_0	0.4	7.8	7.8
	F_1	0.00	10.6	10.6
Seedling leaf length	F_0	3.7	14.1	14.1
	F_1	0.00	13.1	13.1

Reference

Brooks, M.E.; Kristensen, K.; van Benthem, K.J.; Magnusson, A.; Berg, C.W.; Nielsen, A.; Skaug, H.J.; Mächler, M.; Bolker, B.M. GlmmTMB balances speed and flexibility among packages for zero-inflated generalized linear mixed modeling. R J. 2017, 9, 378–400. https://doi.org/10.32614/RJ-2017-066.
